# Estimates of influenza‐associated hospitalisations in tropical Singapore, 2010‐2017: Higher burden estimated in more recent years

**DOI:** 10.1111/irv.12676

**Published:** 2019-08-21

**Authors:** Yixiang Ng, Lily Ai Vee Chua, Stefan Ma, Vernon Jian Ming Lee

**Affiliations:** ^1^ Epidemiology and Disease Control Division Ministry of Health Singapore City Singapore; ^2^ Communicable Diseases Division Ministry of Health Singapore City Singapore

**Keywords:** burden, generalized additive model, hospitalisation, influenza, pneumonia, Singapore, tropics

## Abstract

**Background:**

We previously estimated Singapore's influenza‐associated hospitalisation rate for pneumonia and influenza (P&I) in 2010‐2012 to be 29.6 per 100 000 person‐years, which corresponds to 11.2% of all P&I hospitalisations.

**Objectives:**

This study aims to update Singapore's estimates of the influenza‐associated pneumonia and influenza (P&I) hospitalisation burden using the latest data from 2010 to 2017.

**Methods:**

We estimated the number of P&I hospitalisations associated with influenza using generalised additive models. We specified the weekly number of admissions for P&I and the weekly influenza positivity in the models, along with potential confounders such as weekly respiratory syncytial virus (RSV) positivity and meteorological data.

**Results:**

In 2010‐2017, 16.3% of all P&I hospitalisations in Singapore were estimated to be attributed to influenza, corresponding to an excess influenza‐associated P&I hospitalisation rate of 50.1 per 100 000 person‐years. Higher excess rates were estimated for children aged 0‐4 years (186.8 per 100 000 person‐years) and elderly aged ≥ 65 years (338.0 per 100 000 person‐years). Higher influenza‐associated hospitalisation rates were estimated for 2016 and 2017 (67.9 and 75.1 per 100 000 persons, respectively) years when the influenza A(H3N2) subtype was dominant.

**Conclusion:**

Influenza burden in Singapore has increased since 2010. Influenza vaccination programmes should continue to be prioritised for the young and the elderly.

## 1 INTRODUCTION

There is a widespread consensus that influenza imposes a considerable burden on public health, including substantial numbers of severely ill patients resulting in hospitalisations and deaths.^1^, ^2^, ^3^, ^4^, ^5^, ^6^, ^7^, ^8^, ^9^, ^10^, ^11^, ^12^, ^13^, ^14^, ^15^, ^16^, ^17^, ^18^, ^19^ The impact of influenza on public health varies across seasons, mainly due to the varying influenza virus types/subtypes, vaccine uptake in the community and the match between the recommended vaccine with the circulating viruses.^4^, ^5^, ^6^, ^7^, ^8^ Quantifying the burden of influenza disease is important so as to assess the severity of the virus, evaluate how well public health services are coping with the additional burden or determine whether additional interventions should be considered to reduce the impact.

Estimating the burden is challenging because many who are ill typically receive empiric treatment without laboratory confirmation of influenza infection. In 2015, the World Health Organization (WHO) published a manual to introduce methods to estimate the burden associated with seasonal influenza.^20^ There have also been advances in methods introduced by various studies to measure influenza burden,^21^ including statistical regression models such as the generalised additive model (GAM). An advantage of using regression models is that confounding factors, such as temperature or long‐term time trends, which may influence the observed outcome of choice (ie, pneumonia admissions) can be controlled for.

Singapore, being a small globally connected city‐state in an equatorial region, has a climate that is high in temperature and humidity. It also experiences influenza activity that is vastly different from temperate countries, characterised by year‐round influenza activity and bimodal peaks that usually occur in the beginning and middle of the year.^22^ Previous local studies have highlighted the significant burden that influenza imposes on public health. One older study estimated the influenza‐associated excess mortality rate to range from a low of 0.2 per 100 000 persons in 1975 to a high of 54.4 per 100 000 persons in 1957 during the pandemic.^13^ More recent studies estimated the excess mortality rate for all‐cause deaths to be 14.8 per 100 000 person‐years in 1996‐2003 and the excess hospitalisation rate for pneumonia and influenza to 29.6 per 100 000 person‐years in 2010‐2012.^14^, ^15^ The excess hospitalisation rate was also found to be substantially greater in the very young (infants aged 0‐5 months) and the very old (≥75 years). This study aims to update Singapore's estimates of the influenza‐associated pneumonia and influenza (P&I) hospitalisation burden using the latest data from 2010 to 2017.

## 2 METHODS

### 2.1 Data

We obtained the weekly number of admissions for pneumonia and influenza (P&I) (ICD‐9 480‐487 and ICD10 J10‐J18) for all acute hospitals from the inpatient administrative database. Five age groupings were analysed in our study: 0‐4 years, 5‐49 years, 50‐64 years, ≥65 years and all ages. These age groupings were in accordance with the WHO manual for estimating influenza burden.^20^

The Ministry of Health (MOH), Singapore, has a national surveillance programme for influenza. Virological surveillance is carried out on respiratory specimens from patients with ILI symptoms who visit public primary care centres (accounting for ~ 20% of all primary care consults nationwide),^23^ public hospitals (accounting for ~ 75% of all hospitalisations nationwide)^24^ or sentinel private primary care clinics. These specimens are sent to the National Public Health Laboratory (NPHL) to be tested for influenza and other respiratory pathogens. We used the all‐age weekly influenza positivity from 2010 to 2017, derived by calculating the proportion of specimens that had been laboratory‐confirmed positive for influenza, as a proxy variable for influenza activity. We also highlighted the dominant influenza type/subtype for each year based on results from another study.^25^ To adjust for potential confounding, we also obtained the weekly respiratory syncytial virus (RSV) positivity data from the paediatric departments of two government acute hospitals, as well as meteorological data (weekly mean temperature and weekly mean relative humidity) from the National Environment Agency (NEA). These data sources have been described in more detail in previous studies.^14^, ^15^

### 2.2 Statistical analysis

We used a generalised additive negative binomial model to estimate the weekly number of P&I hospitalisations associated with influenza during the 8‐year study period. Generalised additive models (GAMs) have been used in multiple studies for estimating influenza burden.^4^, ^8^, ^16^, ^17^ An advantage of the GAM is that it allows the data to suggest an appropriate functional form for the relationship between an explanatory variable and the response using a penalised likelihood function.^26^ We also included a spline function to account for the non‐linear long‐term time trend. Each model for the five different age groupings was fitted as follows:

$$\begin{array}{cc} & \text{Log}\;\text{[E(Weekly}\;\text{number}\;\text{of}\;P\&I\;\;\text{hospitalisations)]}\\ & \;=s(t)+\text{s(temperature)}+\text{s(humidity)}+\text{RSV}+\text{Influenza}\;\text{positivity,} \end{array}$$

where s() refers to a cubic smoothing spline function and *t* refers to the week index (1 to 417). To determine the amount of smoothing required for the time trend, temperature and humidity, we examined the relationship between P & I hospitalisations and each explanatory variable by means of a scatterplot smoother. In our model, we used the cubic regression smoothing spline which is versatile in fitting a variety of complex relationships. Over‐fitting or under‐fitting by these cubic smoothing splines would result in under‐estimation or over‐estimation of the influenza burden, respectively. We used a combination of graphical methods and judgement to determine a reasonable fit for each smoothing spline. A smoothing spline of 4 degrees of freedom (*df*s) per year was used for the time trend. For temperature and humidity, we specified a DF value of 3 for both variables. We also tested the significance of lag variables for RSV and influenza positivity (up to 1‐week lag). After fitting the model, we assessed the autocorrelation and partial autocorrelation values (ACF & PACF) of the residuals. The values were within 0.3, which suggested that the temporal trend had been adequately explained (Figure S1).^27^

In the previous model,^15^ we had modelled the time trend and seasonality using a non‐linear variable for week of the year (e‐weeks 1 to 52/53) and a dummy variable for e‐year. In this model, we used a smoothing spline to model the time trend and variability in seasonality patterns across the entire time series. This change has improved the goodness of fit of the long‐term trend and seasonality, especially after incorporating recent years' data.

The weekly number of excess influenza‐associated hospitalisations was defined as the difference between the predicted P&I hospitalisation number from the model and a counterfactual of the predicted number when the influenza positivity variable was set to zero. The annual excess numbers were calculated by summing the weekly excess numbers for each year. Annual influenza‐associated hospitalisation rates (per 100 000) were calculated by dividing the derived excess influenza‐associated numbers with mid‐year total population estimates. Proportions of P&I hospitalisations attributable to influenza were calculated by dividing the excess numbers with the observed P&I numbers. 95% confidence intervals (CIs) of the annual excess numbers were generated by bootstrapping with 1000 resamples. To further examine the difference in influenza burden between the two bimodal influenza peaks seen in Singapore, we specified two time periods that each encompass the ‘mid‐year’ and ‘end‐to‐start‐of‐year' high activity months, respectively: weeks 14 to 39 (April to September) and weeks 40 to 13 (October to March). We compared the estimated number of influenza‐associated hospitalisations from the all‐age model between the two periods.

All weekly data were represented in epidemiological weeks (e‐weeks). Correlation between weekly P&I hospitalisations and influenza positivity was calculated using the Spearman's correlation coefficient. Statistical significance was set at *P*‐value < .05. All statistical analyses were conducted using the R statistical software^28^ version 3.3.1. The GAMs were performed using the ‘mgcv’ package^26^ in R.

## 3 RESULTS

Singapore experienced an average weekly temperature of 27.9°C (range: 25.6‐30.0°C) and an average weekly relative humidity of 80.8% (range: 63.4%‐92.5%) during the 8‐year study period (Table 1). A weekly average number of 319 P&I hospitalisations was observed, with the majority of patients being the elderly aged 65 years and above. The all‐age P&I hospitalisation rates ranged from a low of 239.2 per 100 000 persons in 2010 to a high of 416.2 per 100 000 persons in 2017 (Table 2). Higher overall hospitalisation rates were observed in the 0‐4 and ≥ 65 years age groups at 775.1 per 100 000 person‐years and 2047.6 per 100 000 person‐years, respectively. For all age groups, higher rates were observed in 2016 and 2017.

**Table 1:** Summary statistics of weekly meteorological and pneumonia and influenza (P&I) hospitalisation data, 2010‐2017

	Mean	SD	Percentiles				
			Min	25th	50th	75th	Max
Meteorological variables							
Temperature (°C)	27.9	0.9	25.6	27.2	27.9	28.5	30.0
Relative humidity (%)	80.8	4.7	63.4	77.7	81.1	84.2	92.5
P&I hospitalisations							
All‐age	319	88	161	257	297	363	792
0‐4 y	33	12	9	25	31	40	82
5‐49 y	55	16	16	44	53	63	125
50‐64 y	47	14	19	37	45	55	118
65 y and above	183	54	86	144	170	209	493

**Table 2:** Age‐specific pneumonia and influenza (P&I) hospitalisation rates (per 100 000 person‐years) and virological surveillance for influenza in Singapore, 2010‐2017

	P&I hospitalisation rates					Influenza surveillance					Correlation analysis	
	All‐age	0‐4 y	5‐49 y	50‐64 y	>65 y	Total no. of specimens tested	Yearly influenza positivity (%)	% influenza A among influenza‐positive specimens	% influenza B among influenza‐positive specimens	Dominant influenza type/subtype(s)	Correlation coefficient^a^	*P*‐value
2010	239.2	650.7	61.7	231.5	1759.2	6971	50.3	78.3	21.7	A(H1N1)pdm09	0.57	**<.01**
2011	264.0	827.7	68.8	226.1	1894.3	2903	40.9	76.4	23.6	A(H1N1)pdm09 and A(H3N2)	0.08	.57
2012	271.8	677.9	71.4	250.8	1910.8	2112	46.3	52.1	47.9	B‐Victoria and B‐Yamagata	0.36	**<.01**
2013	272.2	607.6	64.8	244.7	1933.8	1801	42.1	77.4	22.6	A(H3N2)	0.44	**<.01**
2014	291.9	633.3	62.0	259.7	2050.8	2105	49.3	62.3	37.7	A(H3N2) and B–Yamagata	0.74	**<.01**
2015	316.1	729.6	77.4	275.5	2025.7	1952	42.1	79.8	20.2	A(H3N2)	0.47	**<.01**
2016	377.1	935.6	101.6	323.7	2220.5	2263	53.6	63.1	36.9	A(H3N2)	0.58	**<.01**
2017	416.2	1119.4	98.7	350.1	2386.2	1983	51.5	67.3	32.7	A(H3N2)	0.69	**<.01**
Overall	307.7	775.1	75.9	272.4	2047.6	22 090	47.6	71.3	28.7	–	0.36	**<.01**

Bolded values represent signicant results.

a Correlation between weekly all‐age P&I hospitalisation numbers and weekly influenza A&B positivity using the Spearman's method.

**Figure 1:**
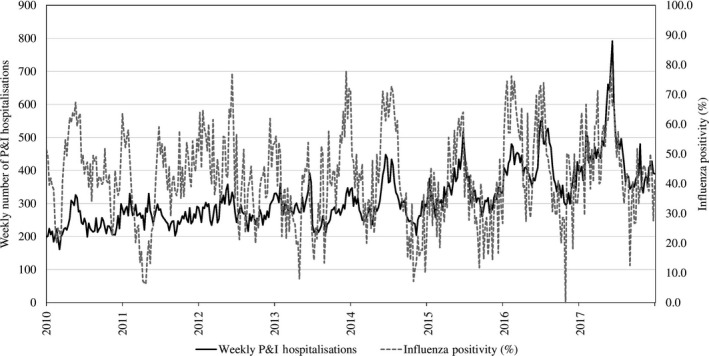
Weekly number of pneumonia and influenza hospitalisations and weekly influenza positivity data, 2010‐2017

A total of 22 090 specimens were tested for influenza during the study period, and the yearly influenza positivity ranged from a low of 40.9% in 2011 to a high of 53.6% in 2016 (Table 2). Every year, more than half of the influenza‐positive specimens were positive for the influenza A subtype. Influenza B also contributed substantially to the influenza‐positive specimens, especially in 2012, 2014, 2016 and 2017 when more than 30% of the influenza‐positive specimens were positive for influenza B. Weekly P&I hospitalisations for all ages were significantly correlated with weekly influenza positivity data for all years except 2011 (*P* = .57). Peaks in weekly P&I hospitalisations generally coincided with the peaks in weekly influenza positivity (Figure 1).

From 2010 to 2017, we estimated the overall influenza‐associated P&I hospitalisation rate to be 50.1 per 100 000 person‐years (95% CI: 48.1‐52.1 per 100 000) (Table 3). Similar to our previous study, the estimated excess rates attributable to influenza were notably different among the age groups. Higher age‐specific excess rates were found for the 0‐4 and ≥ 65 years age groups, at 186.8 per 100 000 person‐years (95% CI: 179.2‐194.9 per 100 000) and 338.0 per 100 000 person‐years (95% CI: 323.9‐352.0 per 100 000), respectively. Approximately 51.2% of influenza‐associated P&I hospitalisations were from the elderly aged ≥ 65. The all‐age excess rate was estimated to be 75.1 per 100 000 persons (95% CI: 67.7‐83.1 per 100 000) in 2017, considerably higher than the estimated rate of 39.9 per 100 000 persons (95% CI: 36.3‐43.6 per 100 000) at the beginning of the study period in 2010.

**Table 3:** Estimated excess rates (per 100 000 person‐years) and its 95% confidence intervals of influenza‐associated pneumonia and influenza hospitalisations by age group, 2010‐2017

	All‐age		0‐4 y		5‐49 y		50‐64 y		>65 y	
	Estimate	95% CI	Estimate	95% CI	Estimate	95% CI	Estimate	95% CI	Estimate	95% CI
2010	39.9	36.3‐43.6	160.8	144.4‐177.8	11.1	10.1‐12.1	55.4	50.6‐60.5	297.1	272.0‐324.3
2011	38.2	33.9‐42.1	171.6	154.0‐187.5	10.6	9.5‐11.7	48.0	42.2‐53.5	279.3	247.3‐308.9
2012	46.3	42.2‐50.9	172.3	156.1‐190.3	13.0	11.9‐14.4	60.7	55.6‐66.5	330.5	301.1‐362.5
2013	40.5	35.5‐45.6	134.0	116.6‐152.3	10.1	9.0‐11.2	52.5	46.2‐58.6	292.5	256.0‐330.0
2014	45.5	38.5‐52.7	148.2	124.0‐173.6	10.2	8.9‐11.5	56.9	48.8‐65.3	325.7	274.6‐376.4
2015	44.7	39.1‐50.3	156.6	135.1‐178.5	11.6	10.4‐12.8	55.6	49.1‐61.9	290.3	254.4‐326.6
2016	67.9	60.4‐74.9	249.4	218.3‐278.8	19.6	17.0‐‐21.9	83.2	74.0‐91.9	403.6	359.8‐445.2
2017	75.1	67.7‐83.1	294.5	265.2‐326.9	19.0	17.0‐20.9	89.4	81.1‐97.9	437.2	394.5‐483.4
Overall	50.1	48.1‐52.1	186.8	179.2‐194.9	13.2	12.7‐13.7	63.3	60.9‐65.7	338.0	323.9‐352.0

Approximately 16.3% (95% CI: 15.6%‐16.9%) of all P&I hospitalisations from 2010 to 2017 were estimated to be attributable to influenza (Table 4). Greater excess proportions were estimated for 2010, 2012, 2014, 2016 and 2017, reflecting the higher influenza positivity percentages. Excess proportions were notably elevated in 2016 and 2017, with estimated excess proportions of 18.0% for both years (95% CI: 16.0%‐19.9% for 2016 and 16.3%‐20.0% for 2017). The proportion of P&I hospitalisations attributable to influenza was found to be highest in the youngest age group of 0‐4 years, with an estimate of 24.1% (95% CI: 23.1%‐25.1%). In contrast, the lowest estimated excess proportion, at 16.5% (95% CI: 15.8%‐17.2%), was found in the elderly aged ≥ 65 years old. All five regression models had moderate to high adjusted R‐squared values (range: 0.66‐0.88), implying that the variation exhibited in the weekly P&I hospitalisation data had been adequately explained.

**Table 4:** Estimated excess proportions and its 95% confidence intervals of pneumonia and influenza hospitalisations attributable to influenza by age group, 2010‐2017

	All‐age		0‐4 y		5‐49 y		50‐64 y		>65 y	
	Estimate	95% CI	Estimate	95% CI	Estimate	95% CI	Estimate	95% CI	Estimate	95% CI
2010	16.7	15.2‐18.2	24.7	22.2‐27.3	17.9	16.3‐19.6	23.9	21.9‐26.2	16.9	15.5‐18.4
2011	14.5	12.8‐15.9	20.7	18.6‐22.6	15.4	13.8‐16.9	21.2	18.7‐23.7	14.7	13.1‐16.3
2012	17.0	15.5‐18.7	25.4	23.0‐28.1	18.2	16.7‐20.1	24.2	22.2‐26.5	17.3	15.8‐19.0
2013	14.9	13.0‐16.7	22.1	19.2‐25.1	15.6	13.9‐17.3	21.4	18.9‐23.9	15.1	13.2‐17.1
2014	15.6	13.2‐18.0	23.4	19.6‐27.4	16.4	14.4‐18.5	21.9	18.8‐25.1	15.9	13.4‐18.4
2015	14.2	12.4‐15.9	21.5	18.5‐24.5	14.9	13.4‐16.5	20.2	17.8‐22.5	14.3	12.6‐16.1
2016	18.0	16.0‐19.9	26.7	23.3‐29.8	19.3	16.7‐21.5	25.7	22.9‐28.4	18.2	16.2‐20.0
2017	18.0	16.3‐20.0	26.3	23.7‐29.2	19.2	17.2‐21.2	25.5	23.2‐28.0	18.3	16.5‐20.3
Overall	16.3	15.6‐16.9	24.1	23.1‐25.1	17.3	16.7‐18.1	23.2	22.4‐24.1	16.5	15.8‐17.2

**Figure 2:**
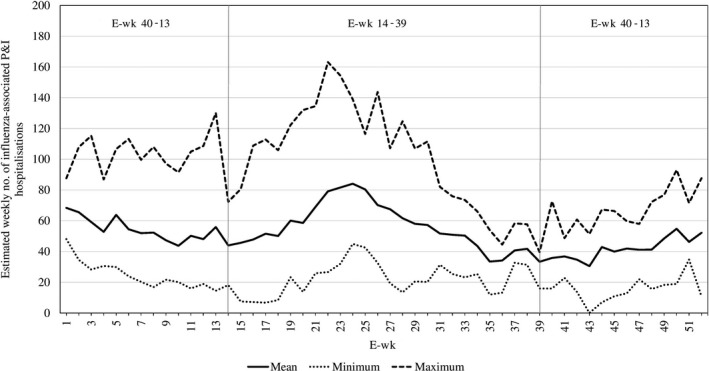
Mean, minimum and maximum estimated number of influenza‐associated pneumonia and influenza hospitalisations from the all‐age model for each e‐week

Influenza burden was found to be higher during the middle of the year. From 2010 to 2017, the mean weekly number of influenza‐associated P&I hospitalisations for the period from weeks 14 to 39 was 55.6, compared to 48.3 for weeks 40 to 13 (*P*‐value from two sample *t* test = .006; Figure 2).

## 4 DISCUSSION

16.3% of all P&I hospitalisations in Singapore from 2010 to 2017 were estimated to be attributed to influenza, corresponding to an excess influenza‐associated P&I hospitalisation rate of 50.1 per 100 000 person‐years. These latest estimates are considerably higher than our previous estimates of 11.2% and 29.6 per 100 000 person‐years using data from 2010 to 2012, and are more reflective of the higher P&I hospitalisations and influenza positivity percentages in recent years. The use of the smoothing spline across the entire time series had improved the model fit and could have explained the difference in estimates from the previous study.

Higher influenza‐associated P&I hospitalisation rates were estimated for 2016 and 2017 years when the influenza A(H3N2) subtype was dominant. The estimated all‐age excess rate of 75.1 per 100 000 persons in 2017 was almost twice the estimated rate in 2010. Age‐standardised rates calculated using the estimated age‐specific rates also showed similar highs in 2016 and 2017 (result not shown). The greater burden estimated for recent years was also discussed in studies conducted by other countries. Neighbouring Indonesia estimated a rate of 19 excess hospitalisations per 100 000 persons at three hospital sites for 2015/2016, higher than past years' estimates.^7^ In the United States, the Centers for Disease Control & Prevention (CDC) estimated an influenza‐associated hospitalisation rate of 185.6 per 100 000 persons during the 2016/2017 season, which was almost double the estimated rate of 95.9 per 100 000 in 2015/2016 but lower than the rate of 221.8 per 100 000 estimated in 2014/2015.^29^ The higher influenza positivity percentages in 2016 and 2017, together with the surge in observed P&I hospitalisation rates, would result in larger estimates for hospitalisations attributable to influenza in Singapore in these two years. 2016 was the first year since 2010 that our local influenza positivity data exceeded 50%, suggesting a resurgence in virus transmissibility since the post‐influenza A(H1N1) 2009 pandemic period.^30^ The increase in observed P&I hospitalisations might be due to the varying composition of respiratory viruses circulating in the community, but it could also be due to an increased susceptibility from an ageing population.

Apart from 2016 and 2017, the years 2010, 2012 and 2014 similarly experienced larger influenza burden based on our all‐age estimates. These five years were characterised by relatively high influenza positivity percentages (46.3% to 53.6%), with four of them (excluding 2010) having higher proportions of influenza B among influenza‐positive specimens (32.7% to 47.9%) compared to other years. Yamagata was the predominant influenza B lineage in 2014 and 2017, while 2012 and 2016 were observed to have co‐circulating lineages with slightly higher proportions of the Victoria lineage.^25^ There is, however, insufficient evidence to conclude that the influenza B virus was driving influenza‐associated hospitalisations in Singapore. One hypothesis is that influenza B could have played an additive effect in the above‐mentioned four years, contributing to the burden generated by influenza A thus leading to greater overall morbidity burden. This could be seen by the higher all‐influenza positivity percentages compared to other years. In other studies, it was commonly found that seasons dominated by the influenza A(H3N2) subtype had typically been associated with higher numbers of influenza‐associated hospitalisations and mortality.^2^, ^18^, ^31^, ^32^ For 2010, the high estimated excess proportions might have been an after‐effect of the 2009 influenza A(H1N1) pandemic^33^ as the population then was probably still building its collective immunity against the emerging influenza strain.

Our overall estimated excess P&I hospitalisation rate of 50.1 per 100 000 person‐years was higher than the average seasonal excess P&I hospitalisation rate of 19.4 per 100 000 persons reported by Portugal from 1998/1999 to 2014/2015, and the overall excess severe acute respiratory infection (SARI) hospitalisation rate of 20.6 per 100 000 persons reported by Oman for 2012‐2015.^5^, ^9^ It was also higher than the annual excess SARI hospitalisation rate of 13‐19 per 100 000 persons from 2013/14 to 2015/16 reported by Indonesia, which has a similar tropical climate. Our estimate was however lower than that of Chile (excess SARI hospitalisation rate of 71.5 per 100 000 person‐years for 2012‐2014), Hong Kong (excess P&I hospitalisation rate of 74.3 per 100 000 person‐years for 1998‐2013) and the United States (average yearly hospitalisations of 133 per 100 000 persons for 2010/2011‐2016/2017).^6^, ^17^, ^29^ As discussed by Abdel‐Hady et al,^5^ it is difficult to make like‐for‐like comparisons between the countries' estimates due to different outcome variables used (ie P&I vs SARI vs all‐cause hospitalisations), unequal health‐seeking behaviour (accessibility to healthcare at an acute hospital), non‐identical methodologies (multiplication method vs statistical regression models) and different study periods. It is notable that given the year‐round influenza activity in Singapore, our annual influenza positivity percentages were consistently more than 40% every year; this is more than double that reported by Chile and Oman (averages of 8.8% and 17%, respectively), countries that experience distinct influenza seasons in the winter.^5^, ^6^ Hence, it could be possible that the burden imposed by influenza on countries with year‐round influenza activity is relatively greater in a calendar year, with all other things being equal.

We estimated higher overall influenza‐associated P&I hospitalisation rates for the young and elderly across the study period, resulting in the J‐shaped curve seen in other studies.^9^, ^15^, ^18^ The higher rates estimated for these two groups suggest that influenza vaccination programmes should continue to be prioritised for the young aged 0‐4 years and the elderly aged 65 years old and above. Vaccination has been found to be one of the most effective ways to reduce influenza burden and also a cost‐effective intervention strategy for targeted age groups.^34^, ^35^, ^36^ Our findings show that influenza burden was higher during the ‘mid‐year’ period (Apr to Sep) compared to the ‘end‐to‐start‐of‐year’ period (Oct to Mar). This corroborates the findings of a previous study showing frequent epidemics and influenza excess mortality during the ‘mid‐year’ period across history.^13^ This shows that we may also have to vaccinate during the middle of the year, in addition to the recommendation by a WHO study to vaccinate in October.^37^ Further studies should be conducted to determine the optimal vaccination schedule in countries with year‐round influenza activity, especially when there is growing evidence of waning vaccine effectiveness.^38^

The estimated proportion of P&I hospitalisations attributable to influenza was the highest for the youngest age group but, in contrast, was the lowest for the oldest age group. These results suggested that while both the young and the elderly were more prone to influenza‐attributed hospitalisations,^10^, ^11^, ^39^ older people were also particularly at risk of developing severe symptoms, and requiring hospitalisations, from other pathogens.

### 4.1 Limitations and strengths

There were a few limitations to our study. Firstly, we used the all‐age influenza positivity variable in all models regardless of age group. Influenza positivity percentages might vary among the different age groups, but the age‐specific weekly number of respiratory specimens tested for influenza was too small for use in a time series model. This was especially so for the 0‐4 years age group as parents could be more reluctant in giving consent for nasopharyngeal swabs to be taken. Likewise, we also used RSV data from paediatrics data in our models due to lack of such routine testing in adults. Secondly, other bacterial and viral causes of pneumonia, such as rhinoviruses and streptococcus pneumonia, were not specifically accounted for in our model. Instead, we used a smoothing spline function to adjust for background variations. Lastly, our study was confined to P&I hospitalisations, which is a subset of all‐cause or respiratory hospitalisations. Hence, our estimates might be an under‐estimation of the true influenza burden.

The strengths of our study were Singapore's comprehensive data collection system and that our observed P&I hospitalisation numbers accounted for all acute hospitals in Singapore. The respiratory specimens tested for influenza were also collected from multiple sites around the country. This means that our data were reflective of the nation as a whole and population‐based estimates were derived.

## 5 CONCLUSION

The overall influenza‐associated P&I hospitalisation rate in Singapore for 2010‐2017 was estimated to be 50.1 per 100 000 person‐years, with higher rates seen in recent years. Higher excess rates were observed in the very young aged 0‐4 years and the elderly aged ≥ 65 years, while the proportion of P&I hospitalisations attributable to influenza was highest in those aged 0‐4 years and the lowest in the elderly aged ≥ 65 years old. Further studies should be conducted to estimate the influenza burden by each subtype.

## References

[1] GBD 2017 Influenza Collaborators . Mortality, morbidity, and hospitalisations due to influenza lower respiratory tract infections, 2017: an analysis for the global burden of disease study 2017. Lancet Respir Med. 2017;7:69‐89. 10.1016/S2213-2600(18)30496-X PMC6302221 30553848

[2] Hayward AC , Fragaszy EB , Bermingham A , et al. Comparative community burden and severity of seasonal and pandemic influenza: results of the Flu Watch cohort study. Lancet Respir Med. 2014;2:445‐454. 24717637 10.1016/S2213-2600(14)70034-7 PMC7164821

[3] Iuliano AD , Roguski KM , Chang HH , et al. Estimates of global seasonal influenza‐associated respiratory mortality: a modelling study. Lancet. 2018;391:1285‐1300. 29248255 10.1016/S0140-6736(17)33293-2 PMC5935243

[4] An der Heiden M , Buchholz U . Estimation of influenza‐attributable medically attended acute respiratory illness by influenza type/subtype and age, Germany, 2001/02‐2014/15. Influenza Other Resp Viruses. 2017;11(2):110‐121. 10.1111/irv.12434 PMC5304576 27754611

[5] Abdel‐Hady DM , Al Balushi RM , Al Abri BA , et al. Estimating the burden of influenza‐associated hospitalization and deaths in Oman, 2012–2015. Influenza Other Respi Viruses. 2018;12:146‐152. 10.1111/irv.12500 PMC5818336 29205882

[6] Sotomayor V , Fasce RA , Vergara N , De la Fuente F , Loayza S , Palekar R . Estimating the burden of influenza‐associated hospitalizations and deaths in Chile during 2012–2014. Influenza Other Respi Viruses. 2018;1‐8. 10.1111/irv.12502 PMC5818356 29446231

[7] Susilarini NK , Haryanto E , Praptiningsih CY , et al. Estimated incidence of influenza‐associated severe acute respiratory infections in Indonesia, 2013–2016. Influenza Other Respi Viruses. 2018;12:81‐87. 10.1111/irv.12496 PMC5818340 29205865

[8] Gul D , Cohen C , Tempia S , Newall AT , Muscatello DJ . Influenza‐associated mortality in South Africa, 2009–2013: the importance of choices related to influenza infection proxies. Influenza Other Respi Viruses. 2017;1‐11. 10.1111/irv.12498 PMC5818357 29197161

[9] Rodrigues E , Machado E , Silva S , Nunes B . Excess pneumonia and influenza hospitalizations associated with influenza epidemics in Portugal from season 1998/1999 to 2014/2015. Influenza Other Respi Viruses. 2018;12:153‐160. 10.1111/irv.12501 PMC5818339 29460423

[10] Cromer D , van Hoek AJ , Jit M , Edmunds WJ , Fleming D , Miller E . The burden of influenza in England by age and clinical risk group: a statistical analysis to inform vaccine policy. J Infect. 2014;68(4):363‐371. 24291062 10.1016/j.jinf.2013.11.013

[11] Matias G , Taylor RJ , Haguinet F , Schuck‐Paim C , Lustig RL , Fleming DM . Modelling estimates of age‐specific influenza‐related hospitalisation and mortality in the United Kingdom. BMC Public Health. 2016;16:481. 27278794 10.1186/s12889-016-3128-4 PMC4898386

[12] Pitman RJ , Melegaro A , Gelb D , Siddiqui MR , Gay NJ , Edmunds WJ . Assessing the burden of influenza and other respiratory infections in England and Wales. J Infect. 2007;54:530‐538. 17097147 10.1016/j.jinf.2006.09.017

[13] Lee VJ , Yap J , Ong J , et al. Influenza excess mortality from 1950–2000 in tropical Singapore. PLoS ONE. 2009;4(12):e8096. 19956611 10.1371/journal.pone.0008096 PMC2779490

[14] Chow A , Ma S , Ling AE , Chew SK . Influenza‐associated deaths in Tropical Singapore. Emerg Infect Dis. 2006;12(1):114‐121. 16494727 10.3201/eid1201.050826 PMC3293465

[15] Ang LW , Lim C , Lee V , et al. Influenza‐associated hospitalizations, Singapore, 2004–2008 and 2010–2012. Emerg Infect Dis. 2014;20(10):1652‐1660. 25275710 10.3201/eid2010.131768 PMC4193272

[16] Guo R‐N , Zheng H‐Z , Ou C‐Q , et al. Impact of influenza on outpatient visits, hospitalizations, and deaths by using a time series poisson generalized additive model. PLoS ONE. 2016;11(2):e0149468. 26894876 10.1371/journal.pone.0149468 PMC4760679

[17] Wu P , Presanis AM , Bond HS , Lau E , Fang VJ , Cowling BJ . A joint analysis of influenza‐associated hospitalizations and mortality in Hong Kong, 1998–2013. Sci Rep. 2017;7:929. 28428558 10.1038/s41598-017-01021-x PMC5430505

[18] Oliva J . Delgado‐Sanz C, Larrauri A, the Spanish Influenza Surveillance System. Estimating the burden of seasonal influenza in Spain from surveillance of mild and severe influenza disease. Influenza Other Respi Viruses. 2018;12:153‐160. 10.1111/irv.12499 PMC5818358 28960828

[19] Gefenaite G , Pistol A , Popescu R , et al. Estimating burden of influenza‐associated influenza‐like illness and severe acute respiratory infection at public healthcare facilities in Romania during the 2011/12‐2015/16 influenza seasons. Influenza Other Respi Viruses. 2018;12:183‐192. 10.1111/irv.12525 PMC5818344 29144598

[20] Global Influenza Program, World Health Organization . A Manual for Estimating Disease Burden Associated with Seasonal Influenza. 2015; Available from: https://apps.who.int/iris/bitstream/10665/178801/1/9789241549301_eng.pdf

[21] Lee VJ , Ho Z , Goh EH , et al. Advances in measuring influenza burden of disease. Influenza Other Respi Viruses. 2018;12:3‐9. 10.1111/irv.12533 PMC5818353 29460425

[22] Ang LW , Tien WS , Lin R‐P , et al. Characterization of influenza activity based on virological surveillance of influenza‐like illness in tropical Singapore, 2010–2014. J Med Virol. 2016;88(12):2069‐2077. 27152935 10.1002/jmv.24566

[23] Ministry of Health, Singapore . Primary care survey 2014 report. Available at https://www.moh.gov.sg/resources-statistics/reports/primary-care-survey-2014-report. Assessed on 4 April 2019.

[24] Ministry of Health, Singapore . Admissions and outpatient attendances. Available at https://www.moh.gov.sg/resources-statistics/singapore-health-facts/admissions-and-outpatient-attendances. Assessed on 4 April 2019.

[25] Ang LW , Lin C , Mak TM , et al. Differential age‐specific distribution of influenza virus types and subtypes based on virological surveillance of influenza‐like illness in tropical Singapore, 2011–2017. J Med Virol. 2019;91(4):1‐8. 30927452 10.1002/jmv.25473

[26] Wood SN . Generalized additive models: an introduction with R (2nd edition). Boca Raton, Florida: Chapman & Hall/CRC;2006.

[27] Hinkle DE , Weirsma W , Jurs SG . Applied Statistics for the Behavioral Sciences (5th edition). Boston: Houghton Mifflin; 2003.

[28] R Core Team . R: A language and environment for statistical computing. R foundation for statistical computing. 2016. Available from: http://ww.r-project.org. Assessed April 18, 2018.

[29] Centers for Disease Control and Prevention . Estimated influenza illnesses, medical visits, and hospitalization averted by vaccination in the United States. 2018 April 2 ; Available from: https://www.cdc.gov/flu/about/disease/2016-17.htm. Assessed January 2, 2019.

[30] World Health Organization . WHO Interim Global Epidemiological Surveillance Standards for Influenza. 2012 July; Available from: https://www.who.int/influenza/resources/documents/INFSURVMANUAL.pdf. Assessed January 3, 2019.

[31] Goldstein E , Viboud C , Charu V , Lipsitch M . Improving the estimation of influenza‐related mortality over a seasonal baseline. Epidemiology. 2012;23(6):829‐838. 22992574 10.1097/EDE.0b013e31826c2dda PMC3516362

[32] Song JY , Cheong HJ , Choi SH , et al. Hospital‐based influenza surveillance in Korea: hospital based influenza morbidity and mortality study group. J Med Virol. 2013;85(5):910‐917. 23508916 10.1002/jmv.23548

[33] World Health Organization . What is the pandemic (H1N1) 2009 virus? 2010 February. Available from: https://www.who.int/csr/disease/swineflu/frequently_asked_questions/about_disease/en/. Assessed January 9, 2019.

[34] Osterholm MT , Kelley NS , Sommer A , Belongia EA . Efficacy and effectiveness of influenza vaccines: a systematic review and meta‐analysis. Lancet Infect Dis. 2012;12:36‐44. 22032844 10.1016/S1473-3099(11)70295-X

[35] Thorrington D , van Leeuwen E , Ramsay M , Pebody R , Baguelin M . Cost‐effectiveness analysis of quadrivalent seasonal influenza vaccines in England. BMC Med. 2017;15:166. 28882149 10.1186/s12916-017-0932-3 PMC5590113

[36] Deans GD , Stiver HG , McElhany JE . Influenza vaccines provide diminished protection but are cost‐saving in older adults. J Intern Med. 2010;267:220‐227. 20175868 10.1111/j.1365-2796.2009.02201.x

[37] Hirve S , Newman LP , Paget J , et al. Influenza Seasonality in the tropics and subtropics – When to vaccinate? PLoS ONE. 2016;11(4):e0153003. 27119988 10.1371/journal.pone.0153003 PMC4847850

[38] Ng Y , Nandar K , Chua L , et al. Evaluating the effectiveness of the influenza vaccine during respiratory outbreaks in Singapore’s long term care facilities, 2017. Vaccine. 2019;37:3925‐3931. 31160102 10.1016/j.vaccine.2019.03.054

[39] Poehling KA , Edwards KM , Weinberg GA , et al. The underrecognized burden of influenza in young children. N Engl J Med. 2006;335:31‐40. 10.1056/NEJMoa054869 16822994

