# Supplementary figures and images for: Estimates of influenza‐associated hospitalisations in tropical Singapore, 2010‐2017: Higher burden estimated in more recent years
Source: Influenza Other Respir Viruses. 2019 Aug 21;13(6):574–81. doi: 10.1111/irv.12676 (PMC6800300; doi:10.1111/irv.12676)

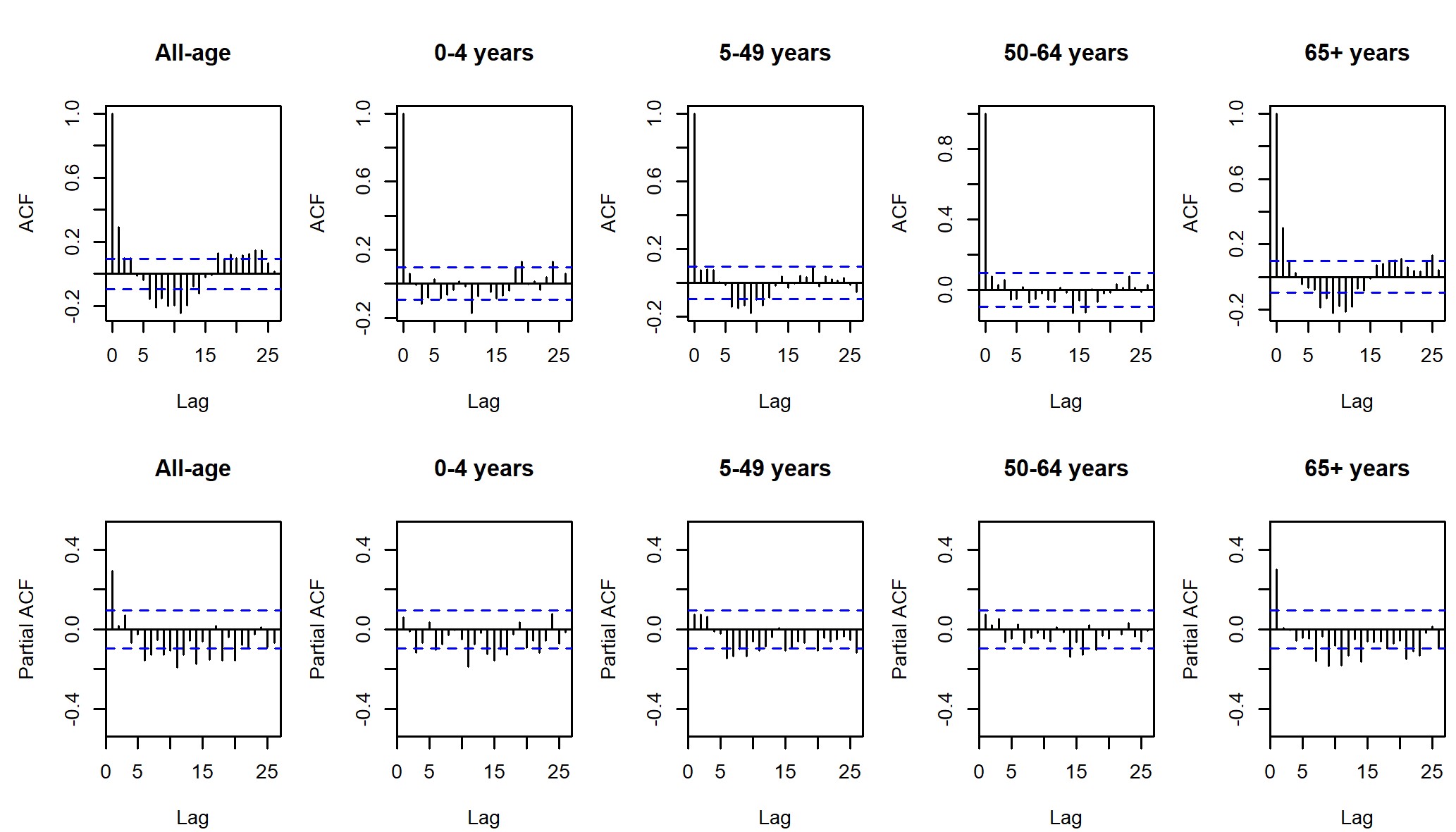

Supplement: Supplementary file 1 [file IRV-13-574-s001.jpg]
